# Effect of multiple intra-articular injections of polynucleotides on treatment of intractable knee osteoarthritis: A case report

**DOI:** 10.1097/MD.0000000000009127

**Published:** 2017-12-08

**Authors:** Jong-Uk Mun, Hyung Rae Cho, Young Soon Choi, Young Uk Kim

**Affiliations:** a Department of Orthopaedic Surgery, Changwon Gyeongsang National University Hospital, Republic of Korea; b Myongji Hospital, College of Medicine, Seonam University, Goyang, Korea; c Department of Anesthesiology and Pain Medicine, Catholic Kwandong University of Korea College of Medicine, International St. Mary‘s Hospital, Incheon, Republic of Korea.

**Keywords:** case report, hyaluronic acid, knee osteoarthritis, polynucleotides

## Abstract

**Rationale:**

Knee osteoarthritis (KOA) is a chronic joint degenerative disease. Intra-articular injection (IAI) of hyaluronic acid (HA) is widely used to treat KOA. However, some HA injections have no effect at all. Polynucleotides (PN) are recently noted as a valid substitute for HA.

**Patient concerns:**

A 61-year-old female was admitted to the pain center with symptoms of pain over the knee and warmth feeling with stiffness in the left knee. The patient reported chronic severe pain in the left knee area despite 6 times IAI of HA. She had past medical history of breast cancer and thyroid cancer.

**Diagnoses:**

She was diagnosed as having KOA.

**Interventions:**

Ultrasound-guided IAI of PN was carried out 3 times in 3 weeks.

**Outcomes:**

She was followed-up for more than 5 months with good improvement in intractable knee pain without any adverse event.

**Lessons:**

IAI of PN is an efficient therapeutic option for KOA treatment if HA injection is unsuccessful.

## 1 Introduction

Knee osteoarthritis (KOA) is a chronic joint disorder characterized by changes in chemical-physico and functional properties of the synovial fluid, degeneration of the articular cartilage, and macroscopical modifications of knee joint.^[1]^ KOA is a leading cause of pain and disability among elderly people.^[2]^ Therapeutic modalities for KOA include analgesics, physiotherapy, intra-articular steroid injection, oral supplementation with chondroitin, glucosamine, nonsteroidal anti-inflammatory drugs (NSAIDs), and topical capsaicin.^[3]^ Intra-articular injection (IAI) of hyaluronic acid (HA) is also commonly used in the treatment of the mid- to moderate-stages of KOA.^[4,5]^ The main effect of HA is a “cushion effect” that reduces the friction of articular cartilage and provides a lubricant composition for intra-articular space. Thus, cartilage is able to maintain elasticity and become more resistant to mechanical stress.^[4,6]^ However, the effect of HA remains still controversial. Previous studies have casted doubt on the disease modifying effect of HA.^[1,7]^ Several adverse events of HA have been reported, including hemarthrosis, synovitis, pseudogout, and muscle pain.^[5]^ Polynucleotides (PN) (product name: Condrotide) has been developed in an attempt to provide nutrients to restore the physiology of the intra-articular environment and articular cartilage homeostasis. PNs are polymer molecules that are able to bind large amounts of water and form a tridimensional gel.^[3]^ Here we report a patient with KOA who underwent ultrasound guided multiple IAI of PN for management of intractable knee pain.

## 2 Case presentation

A 61-year-old female was admitted to the pain center with symptoms of pain over the knee and warmth feeling with stiffness in the left knee. The patient reported chronic severe pain in the left knee area aggravating in the morning. It is exacerbated when she is active. At the time of admission, she walked with a limp due to refractory pain rated 6/10 on visual analog scale (VAS). Physical examination revealed crackling and creaking sound during knee flexion and extension movement. The knee had lost its strength and range of motion. It resisted knee flexion. Her height, weight, and body mass index were 167 cm, 64 kg, and 22.93 kg/m^2^, respectively. She had the past medical history of breast cancer, thyroid cancer, and spinal stenosis. She had received IAI of HA 6 times in 3 months and NSAIDs for intractable pain of the medial knee. However, there was no improvement.

**Figure 1:**
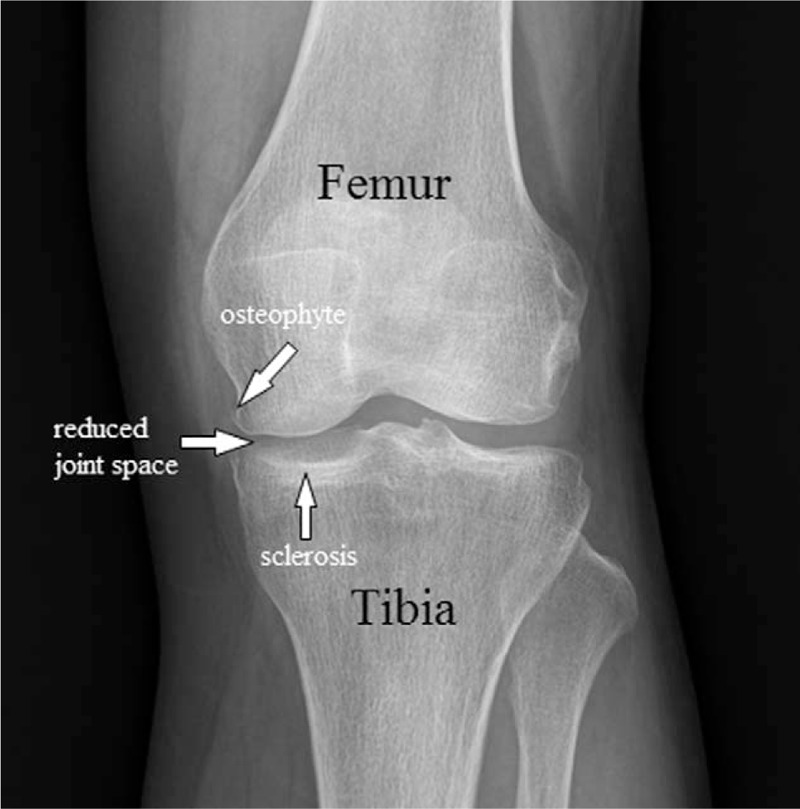
X-ray of the knee showing bone spurs, sclerosis, and a narrowed joint space caused by osteoarthritis.

Knee x-ray revealed the loss of joint space, subchondral sclerosis, and bone spurs developing along edges of the knee joint (Fig. 1).

**Figure 2:**
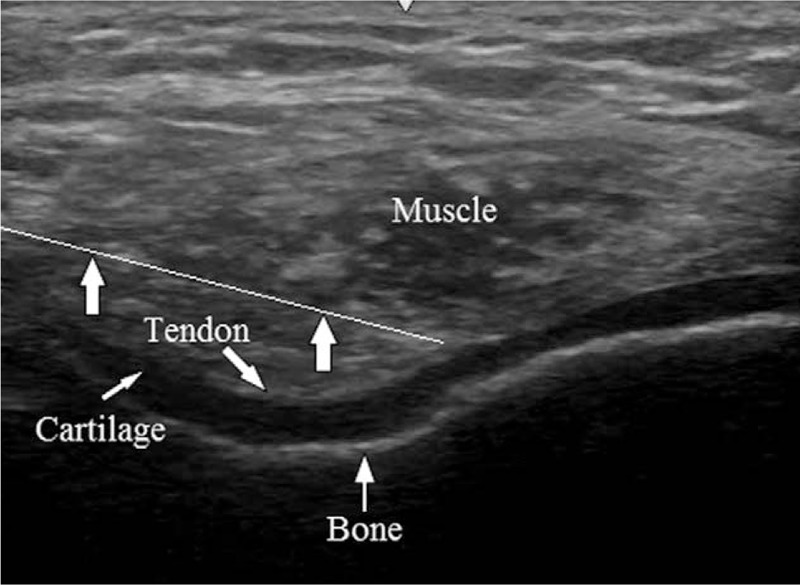
Ultrasound image during injection of polynucleotides in the knee joint space (arrow indicates the block needle.).

We presented therapeutic effects of PN to her. Verbal and written informed consent was obtained from the patient before the procedure. The patient was brought to the ultrasound room and placed on the table in a supine position with bent left knee. We performed ultrasound guided IAI using 40 mg/2 mL of PN with a 26-G 4-cm needle (Fig. 2). At the 1 week follow-up after the IAI of PN, no decrease in VAS score was recorded. The patient reported a persistent refractory pain in her left knee. Therefore, a second IAI of PN was performed. At the 2-weeks follow-up, the patient reported little pain relief with decreased VAS scores from 6 to 5. After the third IAI of PN, she described significant pain reduction with decreased VAS scores from 5 to 1. The patient also showed improvement for all Knee Injury and Osteoarthritis Outcome Score subscales (Table 1).

**Table 1:**
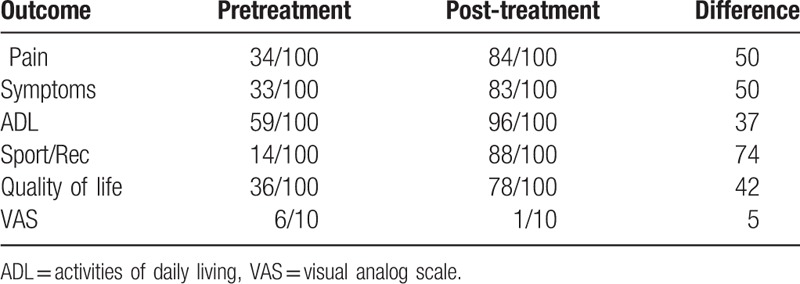
Knee injury and osteoarthritis outcome score and VAS score.

Follow-up was continued for more than 5 months. Clinically, she showed almost complete functional resolution. She was able to jump, run, and hop without disability or pain. She almost completely restored both passive and active ranges of motion without stiffness. We also investigated the adverse effect of PN injection during follow-up. No side-effect was observed.

## 3 Discussion

We present a case of a female patient with KOA who was successfully treated after receiving multiple IAI of PN for intractable knee pain. KOA represents a major burden to public health worldwide. It is the most common subtype of arthritis. The prevalence of KOA is estimated to be 44% in patients over 80 years old and 27% in patients under 70 years old.^[5]^ Progressive stages in KOA are characterized by cartilage erosion, synovial inflammation, subchondral bone sclerosis, increased bone resorption, soft tissue fibrosis, stiffness, and pain in affected joints.^[8–10]^ Diverse pharmacological agents are used for the treatment of KOA, including NSAIDs, IAI of corticosteroid or HA, oral supplements such as chondroitin^[11]^ or glucosamine, and topical treatments such as capsaicin.^[12]^ However, a number of treatments only provide short-term benefit. They may not be satisfactorily effective. Moreover, some procedures are associated with risks. For example, hemarthrosis, synovitis, pseudogout, and muscle pain have been reported after IAI of HA.^[5]^ Autologous conditioned serum, intra-articular mesenchymal stem cell injection, and platelet rich plasma have been recently studied.^[13,14]^ However, currently there are not enough randomized controlled data available. Moreover, these operations are too expensive. Conversely, PNs are polymeric molecules that are subjected to enzymatic cleavage with progressive intra-articular release of both small oligonucleotides (nucleotides, nucleosides, nucleobases) and water molecules. They can deeply moisturize the articular surface, thus maintaining viscoelastic, moisturizing, and metabolic effects for a long time.^[15]^ Giarratana et al^[3]^ have reported a significant reduction in pain after PN injection by maintaining homeostasis of chondrocytes through providing nutritional supplements to the joint and restoring the microenvironment. Vanelli et al^[1]^ have demonstrated that PN can stimulate the healing process of cartilage with remarkable trophic and viscoelastic properties, thus providing satisfactory and stable clinical outcome.

By administering IAI of PN, the synovial fluid is enriched with nucleotides, purine, and pyrimidine bases to support cellular metabolism.^[15]^ Polynucleotides (PN) extracted from fish sperm are highly pure. PNs consist of polymers and polynucleotides ubiquitously present in the human body.^[1]^ Chronic and acute toxicity studies have reported that PN has no toxic effect.^[15]^ Based on safety and the efficacy of PN presented in previous studies, we decided to administer PN and received informed consent from the patient after giving the patient a detailed explanation of the procedure. We conducted ultrasound guided IAI of PN. Despite previous treatments such as IAI of HA, NSAIDS administration, and physiotherapy, they all failed. However, the patient showed good clinical response to PN. The important point of this case report is that IAI of PN is an efficient therapeutic option for treatment of KOA if HA injection is unsuccessful.

## 4 Informed consent

Informed consent was obtained from the patient for publication of this case report and related images.
